# Insertion of Basic Amino Acids in the Hemagglutinin Cleavage Site of H4N2 Avian Influenza Virus (AIV)—Reduced Virus Fitness in Chickens is Restored by Reassortment with Highly Pathogenic H5N1 AIV

**DOI:** 10.3390/ijms21072353

**Published:** 2020-03-28

**Authors:** Marcel Gischke, Reiner Ulrich, Olanrewaju I. Fatola, David Scheibner, Ahmed H. Salaheldin, Beate Crossley, Eva Böttcher-Friebertshäuser, Jutta Veits, Thomas C. Mettenleiter, Elsayed M. Abdelwhab

**Affiliations:** 1 Institute of Molecular Virology and Cell Biology, Friedrich-Loeffler-Institut, Federal Research Institute for Animal Health, Greifswald-Insel Riems, 17493 Mecklenburg-Vorpommern, Germany; Marcel.Gischke@fli.de (M.G.); David.Scheibner@fli.de (D.S.); dr.ahmedhatem@ymail.com (A.H.S.); Jutta.veits@fli.de (J.V.); ThomasC.Mettenleiter@fli.de (T.C.M.); 2 Department of Experimental Animal Facilities and Biorisk Management, Friedrich-Loeffler-Institut, Federal Research Institute for Animal Health, Greifswald-Insel Riems, 17493 Mecklenburg-Vorpommern, Germany; reiner.ulrich@vetmed.uni-leipzig.de (R.U.); Olanrewajulfeoluwa.Fatola@fli.de (O.I.F.); 3 Department of Poultry Diseases, Faculty of Veterinary Medicine, Alexandria University, Alexandria 22758, Egypt; 4 California Animal Health and Food Safety Laboratory, School of Veterinary Medicine, University of California, Davis, CA 95616, USA; bcrossley@ucdavis.edu; 5 Institute of Virology, Philipps University Marburg, 35043 Marburg, Germany; friebertshaeuser@staff.uni-marburg.de

**Keywords:** highly pathogenic avian influenza virus, low pathogenic avian influenza virus, evolution, virulence determinants, non-H5/H7, cleavage site, chicken-to-chicken transmission, virulence, protease

## Abstract

Highly pathogenic (HP) avian influenza viruses (AIVs) are naturally restricted to H5 and H7 subtypes with a polybasic cleavage site (CS) in hemagglutinin (HA) and any AIV with an intravenous pathogenicity index (IVPI) ≥ 1.2. Although only a few non-H5/H7 viruses fulfill the criteria of HPAIV; it remains unclear why these viruses did not spread in domestic birds. In 2012, a unique H4N2 virus with a polybasic CS ^322^PE**KRR**T**R**/G^329^ was isolated from quails in California which, however, was avirulent in chickens. This is the only known non-H5/H7 virus with four basic amino acids in the HACS. Here, we investigated the virulence of this virus in chickens after expansion of the polybasic CS by substitution of T^327^R (^322^PE**KRRRR**/G^329^) or T^327^K (^322^PE**KRRKR**/G^329^) with or without reassortment with HPAIV H5N1 and H7N7. The impact of single mutations or reassortment on virus fitness in vitro and in vivo was studied. Efficient cell culture replication of T^327^R/K carrying H4N2 viruses increased by treatment with trypsin, particularly in MDCK cells, and reassortment with HPAIV H5N1. Replication, virus excretion and bird-to-bird transmission of H4N2 was remarkably compromised by the CS mutations, but restored after reassortment with HPAIV H5N1, although not with HPAIV H7N7. Viruses carrying the H4-HA with or without R^327^ or K^327^ mutations and the other seven gene segments from HPAIV H5N1 exhibited high virulence and efficient transmission in chickens. Together, increasing the number of basic amino acids in the H4N2 HACS was detrimental for viral fitness particularly in vivo but compensated by reassortment with HPAIV H5N1. This may explain the absence of non-H5/H7 HPAIV in poultry.

## 1. Introduction

Influenza A viruses are members of the family Orthomyxoviridae and divided into equine, classical swine/human, gull, bat and avian influenza virus (AIV) lineages [1,2]. The genome of AIVs consists of eight segments coding for at least 10 proteins in addition to strain specific non-structural accessory proteins [1,3]. Based on the antigenic properties of the surface glycoproteins, AIVs are currently classified into 16 hemagglutinin (HA) (H1–H16) and 9 neuraminidase (NA) (N1–N9) subtypes which have been isolated from aquatic and domestic birds in different HxNy combinations [4]. AIVs are further classified according to virulence in chickens into low pathogenic (LP) and highly pathogenic (HP) pathotypes. LPAIV induce only mild or no clinical signs, while HPAIVs cause severe illness with mortality rates up to 100% within a few days [5]. HPAIVs evolve from LP progenitors after circulation in domesticated birds and are naturally restricted to H5 and H7 subtypes. The transition of H5 and H7 viruses from LP to HP is accompanied by mutations due to the error-prone RNA-dependent RNA-polymerase (RdRp) and/or reassortment, i.e., acquisition of gene segments from other subtypes [6]. The HA protein is synthesized as a fusion-inactive precursor (HA0) that requires processing by the host or bacterial proteases into HA1 and HA2 polypeptides at the proteolytic cleavage site (CS). Alteration of the CS from a monobasic to a polybasic motif after insertion of basic amino acids (AAs) arginine (R) and/or lysine (K) is a major virulence factor [7,8]. The monobasic CS of LPAIV is activated by trypsin-like proteases, which are restricted to the respiratory and/or gastrointestinal tracts of birds resulting in only local infections. Human airway trypsin-like protease (HAT) and transmembrane protease serine 2 (TMPRSS2) present in human airways have been shown to cleave HA with a monobasic cleavage site. However, it remains to be investigated whether orthologous proteases (e.g., HAT and TMPRSS2) of birds also support HA cleavage in birds. Conversely, the polybasic CS of HPAIV is cleaved by ubiquitous, subtilisin-like proteases causing systemic infection and multiorgan dysfunction [9].

Despite carrying polybasic CS motifs, some H5 and H7 viruses exhibited low virulence in chickens [6,10]. The virulence of several of these viruses was enhanced by increasing the numbers of basic AAs in the CS or by additional mutations in the HA or other gene segments [11,12,13]. Interestingly, a few natural non-H5/H7 viruses also fulfill the criteria of HPAIV. Several H10Nx viruses with monobasic CS exhibit an intravenous pathogenicity index (IVPI) higher than 1.2 resembling the HPAIV H5/H7 [14,15,16]. Moreover, in August 2012, an H4N2 virus was isolated from quails in California that possessed the polybasic CS motif ^322^PEKRRTR/G^329^ [17]. It is the only non-H5/H7 virus with four basic AAs in the CS, which complies with the HPAIV furin-specific cleavage motif R-X-X-R. Although this virus replicated and transmitted efficiently in chickens, it did not cause morbidity or mortality [17]. It has been reported that some H5/H7 viruses possessed “intermediate” polybasic CS, which evolved stepwise to accumulate an increasing number of basic AAs, due to strand slippage induced by RdRp [18] or a predisposing RNA secondary structure [19], as found in HPAIV H5N2 in Mexico in 1994 [20], H7N7 in Chile in 2002 [21], H7N7 in Canada in 2004 [22] and even the circulating H5N1 Goose Guangdong (Gs/Gd) virus since 1996/1997 [23]. Also, an increase in the number of basic AA in the CS of several H5 LPAIVs with a K/R-K-K/T-R sequence, similar to the current H4N2 virus, resulted in their transformation into HP phenotypes [13,24,25]. Therefore, there is a possibility that this H4N2 virus acquires single mutations by changing T^327^ to either R or K to produce typical H5/H7 HPAIV R-X-R/K-R motifs [26]. It has been shown that non-H5/H7 viruses are capable of shifting to high virulence after acquisition of a polybasic CS [27,28] and other gene segments from HPAIV H5N1 [29]. However, little is known about the resulting impact on viral fitness to explain the lack of expansion of non-H5/H7 HPAIV in birds.

Here, the virulence of the unique H4N2 virus was studied in chickens after the substitution of threonine at position 327 (T^327^) to arginine (R^327^) or lysine (K^327^) to increase the number of basic AAs to five, leading to motifs ^322^PEKRRRR/G^329^ and ^322^PEKRRKR/G^329^. In addition, the impact of reassortment with HPAIV H5N1 or H7N7 on the virulence of H4N2 was investigated.

## 2. Results

Beside the recombinant H4N2_wt specifying CS ^322^PE**KRR**T**R**/G^329^, seven different viruses carrying the H4N2-HA with T^327^R (^322^PE**KRRRR**/G^329^), T^327^K (^322^PE**KRRKR**/G^329^) or (^322^PQ**RRR**G**KKR**/G^331^) combined with the other seven gene segments from H4N2, HPAIV H5N1 or HPAIV H7N7 were successfully generated using reverse genetics (Table 1). Trials to generate an H4N2 carrying a HPAIV H5N2 HACS (^322^PQ**RRRKKR**/G^330^) were not successful, indicating incompatibility with the H4N2_wt. After several rescue attempts, a virus was obtained with a spontaneous insertion of glutamic acid (^322^PQ**RRR**E**KKR**/G^331^) in the HACS.

### 2.1. The Expansion of the Polybasic CS in H4N2 Virus had a Minimal Impact on Virus Replication in Cell Culture but the Replication of the H4N2 Virus Was Significantly Increased by Reassortment with H5N1 or H7N7

**Figure 1:**
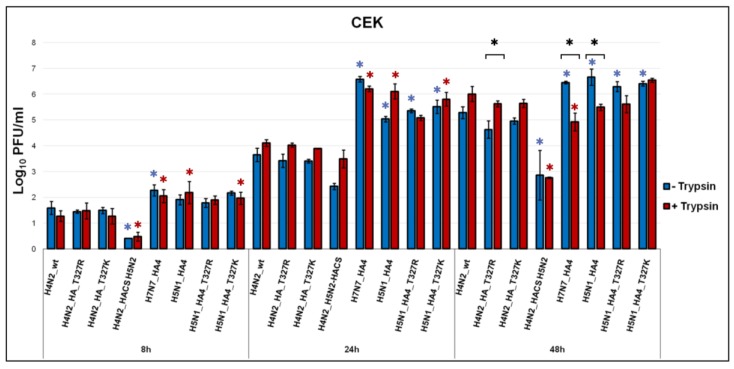
Replication kinetics of recombinant viruses in chicken embryo kidney cells (CEK). Replication kinetics in CEK cells at indicated time points after infection with H4N2 viruses with variable HACS or with H7N7 and H5N1 gene segments with (T+) or without (T−) trypsin. Titration was done in MDCKII cells and the results are shown as mean ± standard deviation Log_10_ PFU/mL. Asterisks indicate significant difference (*p* < 0.05). Blue and red asterisks indicate significant differences compared to H4N2_wt without or with trypsin, respectively. Black asterisks indicate significant differences for replication of each virus in the presence or absence of trypsin.

**Figure 2:**
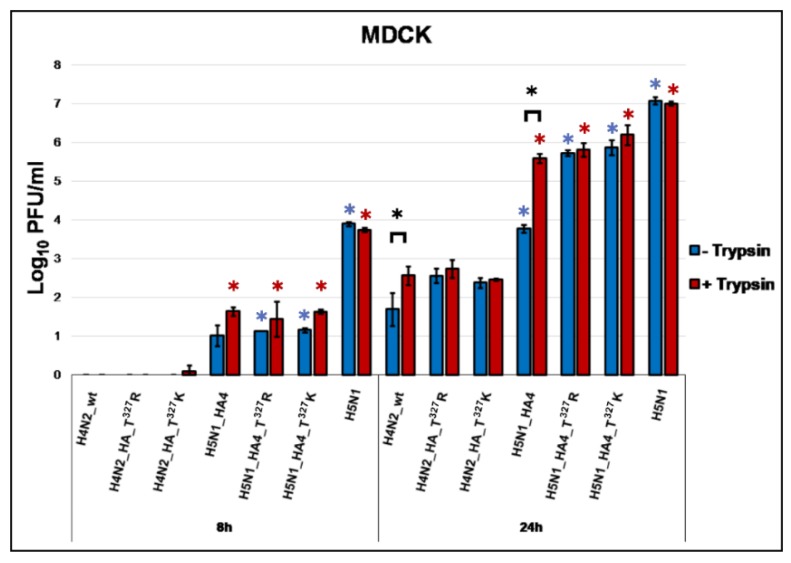
Replication kinetics of recombinant viruses in Madin–Darby canine kidney cells (MDCK). Replication kinetics in MDCK cells at indicated time points after infection with H4N2 viruses with variable HACS or H5N1 gene segments with (T+) or without (T−) trypsin. Titration was done in MDCKII cells and the results are shown as mean ± standard deviation Log_10_ PFU/mL. Asterisks indicate significant difference (*p* < 0.05). Blue and red asterisks indicate significant differences compared to H4N2_wt without or with trypsin, respectively. Black asterisks indicate significant differences for replication of each virus in the presence or absence of trypsin.

Virus replication was studied after the infection of CEK (Figure 1) and MDCK (Figure 2) cells with an MOI of 0.001 in the presence or absence of exogenous trypsin. In CEK, the H4N2_wt replicated with or without trypsin reaching maximum titers at 48 hpi. The addition of trypsin slightly increased virus titers at 24 and 48 hpi, although the difference was not statistically significant (*p* > 0.35) (Figure 1). Replication of H4N2_T^327^R and H4N2_T^327^K with a point mutation in the HA was comparable to H4N2_wt irrespective of the presence of trypsin at 8 and 24 hpi (Figure 1), while the addition of trypsin significantly increased H4N2_T^327^R replication at 48 hpi (*p* < 0.03) (Figure 1). Replication of H4N2_H5N2-HACS was significantly reduced compared to H4N2_wt (Figure 1). H7N7_HA4 carrying the HA from H4N2_wt and the other seven gene segments from HPAIV H7N7 replicated to significantly higher levels than H4N2_wt at 8, 24 and 48 hpi (Figure 1). Moreover, the three viruses carrying seven gene segments from HPAIV H5N1 replicated to significantly higher titers than H4N2_wt particularly at 24 hpi, however, expansion of the HACS did not alter the replication level in this panel of reassortments in CEK (Figure 1). In MDCK cells, H4N2 viruses carrying authentic HA, HA_T^327^R or HA_T^327^K showed no replication at 8 hpi without trypsin and only H4N2_HA_T^327^K produced viral progeny at very low titers in the presence of trypsin (Figure 2). H4 viruses carrying gene segments from H5N1 were able to replicate trypsin-independently but replication increased after the addition of trypsin. At 24 hpi, all viruses replicated without trypsin while viruses carrying H5N1 gene segments replicated at significantly higher titers than H4N2 viruses with or without T^327^R/K. Furthermore, trypsin increased the replication of H5N1_HA4 to similar levels as H5N1_T^327^R/K (Figure 2). Moreover, the H5N1 virus replicated in MDCK cells at higher levels than H4N2 viruses and trypsin had no significant impact on H5N1 virus replication (*p* > 0.49). Together, the replication of H4N2 viruses was enhanced by the addition of trypsin particularly in MDCK cells and reassortment with HPAIV H5N1 or H7N7 segments in CEK.

### 2.2. Cell-to-Cell Spread of H4N2 Virus Was Significantly Increased by Reassortment with H7N7 or H5N1 Containing HA4_T^327^R/K

**Figure 3:**
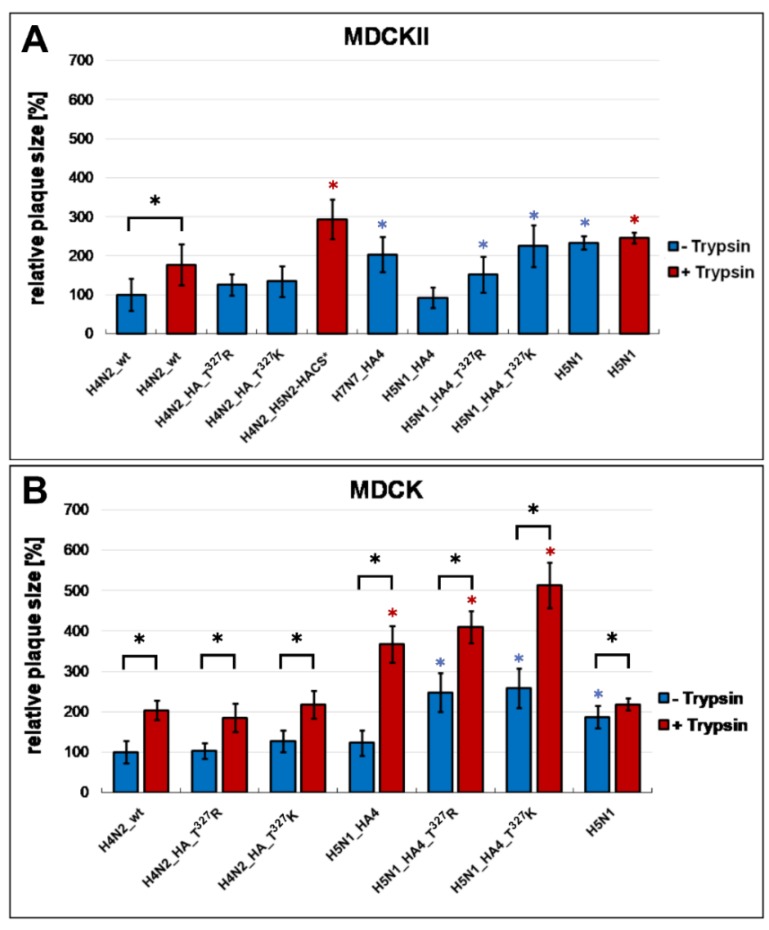
Cell-to-cell spread in MDCKII and MDCK cells. Cell-to-cell spread was assessed by measuring 50 to 100 plaques in MDCKII (**A**) or MDCK (**B**) cells with (T+) or without (T–) trypsin. In MDCKII (**A**), trypsin was added only to H4N2_wt and H4N2_H5N2-HACS. The latter virus did not produce plaques without trypsin. Results are expressed as mean and standard deviation relative to the plaque size of H4N2_wt in the absence of trypsin. Asterisks indicate significant differences (*p* < 0.05); blue and red asterisks indicate significant differences compared to H4N2_wt without or with trypsin, respectively. Black asterisks indicate significant differences for the replication of each virus in the presence or absence of trypsin.

Cell-to-cell spread was studied by infecting MDCKII and MDCK cells with different virus dilutions for three days. In MDCKII cells, all viruses were tested without the addition of trypsin except for H4N2_wt and H4N2_H5N2-HACS. The H4N2_wt virus produced plaques in MDCKII irrespective of the presence of trypsin, although the addition of trypsin significantly increased the size of plaques (Figure 3A). The spread of H4N2_T^327^R/K or H5N1_HA4 from cell-to-cell was comparable to H4N2_wt in the absence of trypsin. However, H4N2_H5N2-HACS produced significantly smaller plaques without trypsin while in the presence of trypsin they were larger than those induced by H4N2_wt (Figure 3A). The plaque size of H4N2_T^327^R or H4N2_T ^327^K significantly increased by 52% and 125% in combination with the other seven H5N1 segments (Figure 3A). In MDCK cells, the addition of trypsin increased the plaque size of all viruses. Viruses carrying H5N1 segments produced larger plaques than H4N2_wt, particularly when grown in medium containing trypsin (Figure 3B). Furthermore, the H5N1 virus produced significantly larger plaques in MDCKII and MDCK cells compared to H4N2_wt particularly in the absence of trypsin. Notably, trypsin increased cell-to-cell spread of all viruses in MDCK cells. Taken together, the expansion of the polybasic CS had a minimal impact on virus spread in cell culture but the replication of H4N2 virus was significantly increased by reassortment with H5N1 and/or the addition of trypsin.

### 2.3. T^327^R/K Enabled Partial Trypsin-Independent Activation of the HA by Endogenous Furin-Like Protease

**Figure 4:**
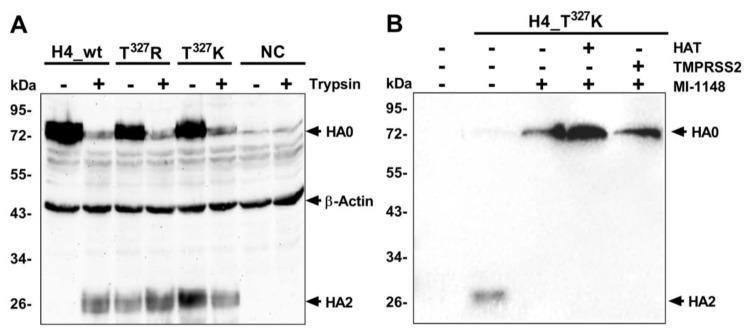
Cleavability of the HA after transfection of HEK293T cells. Cleavability of the HA of H4N2_wt or HA with R^327^or K^327^ in the presence or absence of exogenous trypsin tested with a Western Blot using β-Actin as internal controls (**A**). The cleavability of HA of H4N2_T^327^K in HEK293T cells by TMPRSS2, HAT or furin-like proteases in the presence or absence of the MI-1148 furin inhibitor. No HA2 bands were detected indicating a lack of activation of HA0 by TMPRSS2 and HAT (**B**). NC refers to the negative control; naïve cells without transfection or infection.

To determine the impact of expansion of the CS on HA cleavage, HEK293T cells were transfected with pCAGGS plasmids containing HA_wt, T^327^R or T^327^K with or without trypsin. The wild-type HA was only cleaved in the presence of trypsin. HAs with T^327^R/K were partially cleaved in the absence of trypsin but the addition of trypsin increased cleavability (Figure 4A). Furthermore, HEK293T cells were co-transfected with plasmids encoding HAT and TMPRSS2 in the presence or absence of the furin-inhibitor MI-1148. In the absence of exogenous protease, T^327^K was still cleaved. The cleavage of HA was inhibited using MI-1148. HAT and TMPRSS2 failed to activate HA_T^327^K (Figure 4B). The expression of TMPRSS2 and HAT in HEK293T cells was confirmed using a Western Blot. Altogether, T^327^R/K enabled partial activation of the HA in the absence of trypsin by a yet to be identified endogenous furin-like protease.

### 2.4. The Expansion of the Cleavage Site Alone Was Not Enough for Exhibition of High Virulence after ON (Oculonasal) Inoculation and Reassortment with HPAIV H5N1 Genes Was Essential

After ON inoculation, chickens challenged with H4N2_wt, H4N2_T^327^R, H4N2_T^327^K, H4N2_H5N2-HACS and H7N7_HA4 and contacts did not show any clinical signs with a PI of 0 (Table 2). All experimentally infected birds in these groups seroconverted at the end of the experiment, except for one chicken inoculated with H4N2_H5N2-HACS (Table 2). While all sentinels in-contact with H4N2_wt, H4N2_T^327^R, and H7N7_HA4 ON-inoculated chickens seroconverted, only 1/4 and 0/4 sentinel birds co-housed with chickens ON-inoculated with H4N2_T^327^K or H4N2_H5N2-HACS exhibited seroconversion (Table 2), indicating poor bird-to-bird transmission. Moreover, H5N1_HA4 caused transient mild to moderate clinical signs without mortality (PI = 0.5) and all inoculated and contacts seroconverted (Table 2). All chickens inoculated with H5N1_HA4_T^327^R or H5N1_HA4_T^327^K died within 4 dpi with MDT values of 3.8 and two days, and PI values of 2.4 and 2.7, respectively. Furthermore, 3/4 and 4/4 contact birds died within 8 and 4 dpi, respectively (Table 2).

After IV infection, none of the H4N2_T^327^R injected chickens showed morbidity or mortality with IVPI values of 0. The H4N2_T^327^K group exhibited moderate virulence with an IVPI of 0.6 and moderate to severe neurological disorders (e.g., torticollis, opisthotonos and paresis) starting at 9 dpi were observed in 7 out of 10 injected chickens. The IVPI values of H4N2_H5N2-HACS and H7N7_HA4 were 0.1 and 0.3, respectively, because several chickens developed transient mild depression after injection. Conversely, 8/10 and 10/10 chickens injected IV with H5N1_HA4 or H5N1_HA4_T^327^K died with IVPI 2.1 or 2.8, respectively (Table 2). These findings indicate that H5N1 gene segments, in addition to mutations in the HACS, are essential for the exhibition of the high virulence of the H4N2 virus. T^327^K and H5N2_HACS compromised virus transmission as indicated by the lower number of contact birds with AIV antibodies.

### 2.5. Virus Excretion in Inoculated and In-Contact Chickens Was Reduced by T^327^R/K and Increased by Reassortment with HPAIV H5N1 Segments

**Figure 5:**
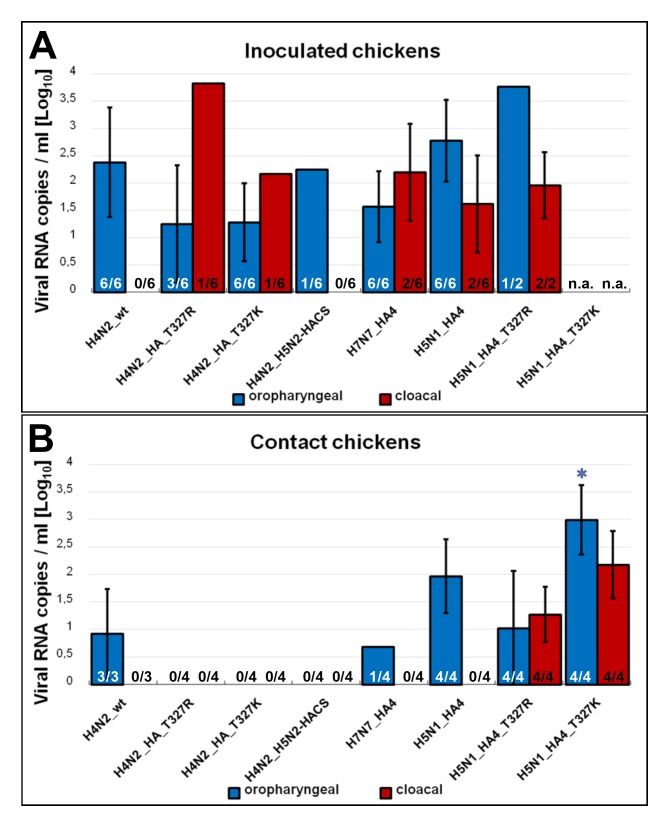
Virus excretion from oropharyngeal and cloacal swabs of inoculated and sentinel chickens. Virus excretion in oropharyngeal and cloacal swabs in inoculated (**A**) and contact (**B**) birds was determined by RT-qPCR targeting the M gene. The averages ± standard deviation of viral RNA copies/ml and number of positive birds/total examined are shown. Samples were collected at 4 dpi from all surviving birds. n.a. = not applicable because all birds inoculated with H4N2_T^327^K died at 2 dpi.

The H4N2_wt was detected at 4 dpi in oropharyngeal, but not in cloacal, swabs in all inoculated and contact birds (Figure 5). H4N2_T^327^R and H4N2_T^327^K were excreted in 3/6 and 6/6 in oropharyngeal swabs, respectively and only in 1/6 cloacal swabs in inoculated birds (Figure 5A). Both viruses were not detected in swabs in contact birds further indicating the negative impact on virus transmission (Figure 5B). Likewise, H4N2_H5N2-HACS RNA was only detected in the oropharyngeal swabs taken from 1/6 inoculated chickens indicating insufficient replication and bird-to-bird transmission (Figure 5A,B). Moreover, H7N7_HA4 RNA was detected in oropharyngeal and cloacal swabs in 6/6 and 2/6 inoculated birds, respectively, and only 1/4 contact bird excreted virus in the oropharyngeal swabs (Figure 5). H5N1_HA4 RNA was detected in 6/6 and 2/6 oropharyngeal and cloacal swabs of inoculated birds, respectively, and all contact birds excreted virus in oropharyngeal swabs (*n* = 4/4) but not in cloacal swabs (*n* = 0/4) (Figure 5A,B). H5N1_HA4_T^327^R was detected in oropharyngeal (*n* = 1/2) and cloacal (*n* = 2/2) swabs (Figure 5A). As H5N1_HA4_T^327^K killed all inoculated birds within two days, it was not possible to collect swabs at 4 dpi in this group. Both H5N1_HA4_T^327^R and H5N1_HA4_T^327^K were excreted from all contact birds (Figure 5B). H5N1_HA4_T^327^K had significantly higher titers in the oropharyngeal swabs compared to H4N2_wt (Figure 5). In summary, mutations in the CS alone compromised virus excretion from inoculated and in-contact chickens. Reassortment with segments from HPAIV H5N1 increased virus excretion in oropharyngeal and cloacal swabs.

### 2.6. Mutation T^327^R/K Expanded the Organ Tropism of LPAIV H4N2 and Reassortment with HPAIV H5N1 Significantly Increased the Distribution and Severity of Lesions

**Figure 6:**
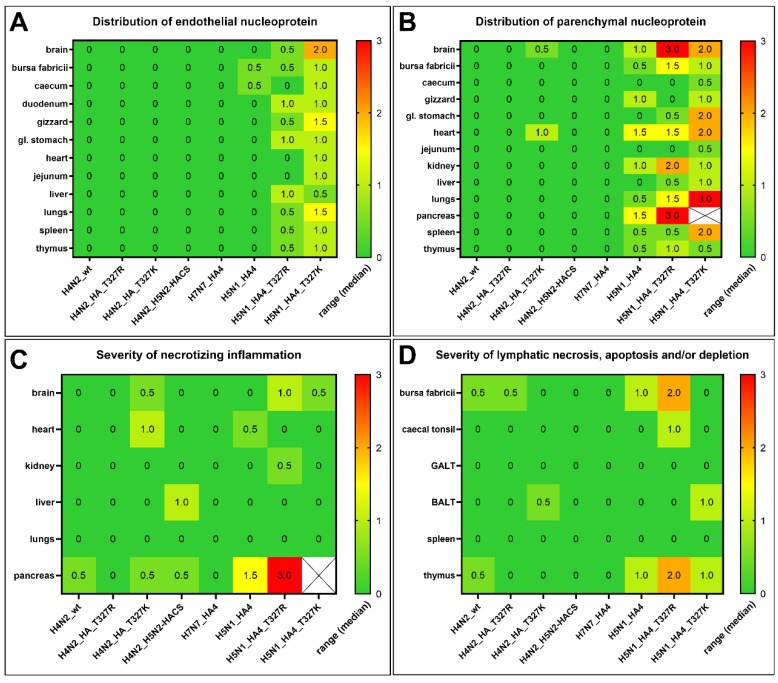
Distribution of avian influenza virus NP in organs of inoculated birds and severity of lesions. Distribution of NP antigen in endothelial (**A**) and parenchymal (**B**) cells as well as the severity of necrotizing inflammation (**C**) and lymphatic depletion (**D**) in the affected organs of inoculated birds scored from 0 to 3.0 (green to red color). Results are shown as the median score of two birds. Samples were collected at 4 dpi for all chickens, except H5N1_H4_T^327^K inoculated birds, which died at 2 dpi and were kept in the refrigerator until 4 dpi. Pancreas samples with white cells and an X symbol in panels B and C were not tested because of insufficient quality for evaluation.

**Figure 7:**
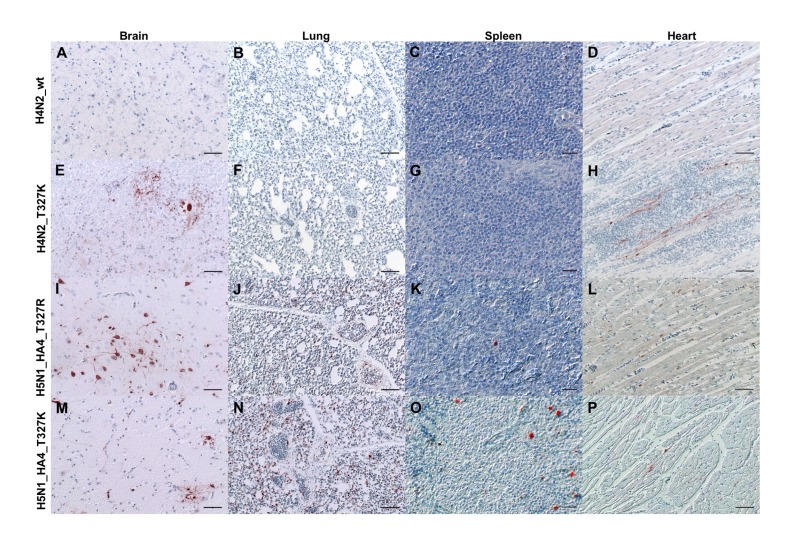
Distribution of avian influenza virus NP in selected organs of inoculated birds. Distribution of influenza NP in brain (**A**,**E**,**I**,**M**), lung (**B**,**F**,**J**,**N**), spleen (**C**,**G**,**K**,**O**) and heart (**D**,**H**,**L**,**P**) of inoculated chickens of selected viruses at 4 dpi (except for H5N1_H4_T^327^K) as detected by immunohistochemistry using primary polyclonal rabbit anti-NP A/FPV/Rostock/34 antibody (1:750) and a secondary biotinylated goat anti-rabbit IgG (Vector Laboratories, Burlingame, CA, USA) antibody (1:200). 3-amino-9-ethyl-carbazol (red-brown); hematoxylin counterstain (blue); Nomarski contrast; bars A,B,D,E,F,H,I,J,L,M,N,P = 20 µm. Bars C,G,K,O = 50 µm.

To determine virus distribution in different tissues, organs of at least two inoculated chickens per group were subjected to histopathological and immunohistological examination for the detection of the influenza NP antigen. There was no detectable antigen in the endothelium or parenchyma of any organ in birds inoculated with H4N2_wt, although mild, subacute, necrotizing pancreatitis and lymphatic depletion in the thymus and bursa of Fabricius were observed (Figure 6). Likewise, the NP antigen was not detectable in the endothelium or parenchyma of any organ in birds inoculated with H4N2_T^327^R. However, one out of two birds inoculated with H4N2_T^327^K had multifocal antigen distribution in the myocardium with mild, acute, focal to oligofocal necrotizing myocarditis as well as focal to oligofocal distribution in neuroglial cells with mild, acute, focal to oligofocal, necrotizing polioencephalitis (Figure 6 and Figure 7). The distribution of H4N2_T^327^K was more widespread than that of H4N2_T^327^R (Figure 6). Similar to H4N2_wt infection, NP was not detected in the endothelium or parenchyma of any organ in birds inoculated with H4N2_H5N2-HACS or H7N7_HA4 (Figure 6A,B). The reassortment with HPAIV H5N1 remarkably increased the distribution of the virus in different tissues. One out of two birds inoculated with H5N1_HA4 showed coalescing NP-antigen-positive cells in the heart and pancreas, multifocal distribution in the kidney, gizzard and brain, and focal to oligofocal distribution in the parenchyma of thymus, lung, spleen and bursa as well as in the endothelial cells in the cecum and bursa. This bird showed moderate to severe lymphoid depletion with tingible body macrophage hyperplasia in the thymus and mild lymphoid depletion in the bursa. Also, severe, acute, necrotizing pancreatitis and subacute, necrotizing myocarditis were observed. The NP of H5N1_HA4_T^327^K was detected in the endothelial and parenchymal cells of almost all organs (Figure 6A,B) and the intensity ranged from median scores of 0.5 in the hepatic endothelium as well as in the thymus, jejunum and caecum parenchyma to 3.0 in the lung parenchyma. Likewise, H5N1_HA4_T^327^R was detected in the endothelium and parenchyma of almost all organs of at least one chicken, except for endothelial cells in the jejunum, heart and caecum and gizzard parenchyma. The maximal distribution for this virus was in the pancreas parenchyma and brain tissue with a score of 3.0. Remarkably, H5N1_HA4_T^327^R induced a higher lymphatic depletion score in the thymus, bursa, cecal tonsils and bronchus-associated lymphoid tissues (BALT) compared to H5N1_HA4_T^327^K and H5N1_HA4. In conclusion, reassortment with HPAIV H5N1 significantly increased the distribution and severity of lesions. The distribution of viruses carrying T^327^K was more widespread than viruses carrying T^327^R (Figure 6 and Figure 7), except for lymphoid depletion.

## 3. Discussion

Wild birds represent the natural reservoir for LPAIV. HPAIVs evolve from LPAIV of H5 or H7 subtype after the acquisition of a polybasic CS, which is specific in each HPAIV. In 2012, an H4N2 virus with polybasic CS ^322^PEKRRTR/G^329^, closely related to H4N2 viruses with monobasic CS ^322^PEKTR/G^329^ from wild birds in the USA, was isolated from a commercial quail flock in California [17]. In our study, the acquisition by H4N2 virus of a “classical” polybasic CS either by mutation of T^327^ to K^327^ or R^327^, or the substitution by an H5N2-like HACS did not improve virus replication or spread in the absence of trypsin. Conversely, H4N2_H5N2-HACS was highly trypsin dependent as shown by low virus titers in cell culture and restricted cell-to-cell spread. A similar example is the trypsin-dependent Pennsylvanian H5N2/1983 virus with a polybasic CS, which was efficiently cleaved by furin-like enzymes only after the insertion of further basic AAs or removal of a glycosylation site in the vicinity of the CS [30]. Interestingly, the current H4N2 virus has potential glycosylation sites in the vicinity of CS in the HA1 [17], resembling H5N2/1983, which hinder cleavage by different proteases [31,32]. Moreover, we showed that furin-like protease(s) can also cleave the HA of H4N2_T^327^K in transfected HEK293T independent of the presence of HAT and TMPRSS2 that activate some viruses with monobasic CS [33] and some H9N2 viruses with monobasic VSSR/G, dibasic RSSR/G or tribasic RSRR/G cleavage site motifs [32]. Interestingly, these viruses were not activated by furin without further insertion of basic AAs at the CS despite matching the minimal consensus sequence [27,34]. Furthermore, Wong et al. [17] showed that replication or plaque formation of the wild type H4N2 virus in MDCK cells was trypsin dependent. However, the current reverse-engineered H4N2 virus and derivatives induced plaques with variable size in MDCKII or MDCK cells and replicated in CEK without the addition of trypsin. MDCKII cells are a natural subclone of MDCK cells and both cell lines are commonly used for characterization of AIV. It has been reported that MDCKII and CEK cells have matriptase, which is not present in the MDCK cells [32]. Matriptase activated H9N2 AIVs with R-X-X-R or R-X-R-R motifs [32], similar to the HACS of H4N2 viruses generated in this study. Moreover, some LPAIVs (e.g., H6N1 and H7N7) were able to replicate in MDCK, MDCKII and/or CEK cells without exogenous trypsin [28,35]. In addition to the unidentified endogenous proteases in these cells, an impact of proteases in the allantoic fluid in virus stocks on activation in different cells cannot be excluded [36]. Moreover, reassortment with the H5N1 segments increased H4N2 virus spread more effectively in MDCK cells. H4N2_wt possessed E627, while HPAIV H5N1 possessed K627 in PB2 [37], which is known to increase the polymerase activity and replication of H5N1 virus in mammal cells [37].

Successful replication of an AIV in poultry is a prerequisite for progressive adaptation including efficient bird-to-bird transmission and high virulence [38]. We show here that the expansion of the authentic polybasic CS by insertion of K^327^, R^327^ or substitution by an H5N2-like HACS was detrimental for H4N2 virus excretion and bird-to-bird transmission. Therefore, the negative impact of additional basic AAs in the CS on virus replication and transmission in chickens probably precludes their expansion in nature. Moreover, the increased number of basic AAs in the presence of other gene segments from H4N2 did not result in an HP phenotype after ON or IV infections [24,35]. In other studies, the insertion of a polybasic CS conferred high virulence to a low-pathogenic H6N1 virus (IVPI = 1.4) [28] but not an H3N8 virus [39]. Importantly, high virulence of the current H4N2 virus was only conferred after reassortment with gene segments from HPAIV H5N1. These findings emphasize the role of other gene segments, in addition to the polybasic CS, in the evolution of HPAIV [11,12,40]. H9N2 with polybasic CS and gene segments from HPAIV H5N1 exhibited a low-level HP phenotype (IVPI = 1.23) [27]. In contrast, H2N5, H4N6, H8N4 and H14N3 viruses exhibited high virulence after the acquisition of an H5N2-polybasic CS and other gene segments from HPAIV H5N1 [29]. Remarkably, high virulence was not observed after the reassortment of the H4N2 HA with HPAIV H7N7. We have recently shown that the HA gene of this HPAIV H7N7 is the main determinant of virulence in chickens [41]. Conversely, for the current HPAIV H5N1, in addition to the polybasic CS, a deletion within the NA stalk domain (which is also present in the H4N2 virus [17]) and the presence of autologous polymerase genes were important for high virulence in chickens [11]. Furthermore, compared to the H4N2 virus, HPAIV H5N1 used in this study possessed residues in the NP (S377N) [42], NS1 (deletion of AAs 80–84) [43] and PB1 (V14A) [44]. These residues were linked to higher virulence or transmission of H5N1 viruses in chickens, which remain to be investigated. Similarly, it has been also shown that residues, yet to be identified, in PB2, PB1 and NP affect the high virulence of HPAIV H5N1 in chickens [12].

It is known that the presence of a polybasic CS increases the dissemination of HPAIV H5/H7 in different organs causing multiorgan dysfunction and death [7,15,26,28,39]. We showed here that a polybasic CS alone was not sufficient for unrestricted organ tropism and that other gene segments were required particularly to invade the endothelium to vital organs like the brain. This may indicate that the quail virus is less adapted to chicken cells than the panzootic Goose/Guangdong-like H5N1 virus. Apart from the activation of HA by cellular proteases, other gene segments can influence influenza virus activation and replication as well. The NA enhanced the cleavability of the HA of WSN H1N1 and subsequently the neurovirulence of the virus in mice [45,46]. Also, the M2 protein protects the HA from premature conformational changes increasing the stability of influenza viruses [47]. Therefore, it is important to further determine the specific gene segment(s) of H5N1 that support the HP phenotype of H4N2 virus.

Another finding was that T^327^K was advantageous over T^327^R; it increased plaque size, virulence in chickens after IV injection, tropism and excretion from inoculated birds, particularly when combined with H5N1 gene segments. This may indicate cleavage-activation of this CS motif (^322^PEKRR**K**R/G^329^) by additional or more specific furin-like proteases. Some proteases have different preferences for K and R at different positions [48]. For example, lysine in position P2 can greatly enhance the processing efficiency of furin-like enzymes. In one study, 20 (52%) out of the 38 cleavage motifs comply with furin specific sequences were R–X–K–R and 11 (29%) were R–X–R–R giving preferences for lysine over arginine in this position [49]. Intriguingly, the majority of HPAIV H5/H7 possessed lysine at position P2 [23], resembling the T^327^K in this study, which may support our assumption. Remarkably, the pathology of H5N1_HA4_T^327^R was more severe in the bursa, thymus, pancreas and brain compared to H5N1_HA4_T^327^K and H5N1_HA4. This is probably due to the observed higher lymphatic depletion and/or efficient replication of the virus in these organs.

In conclusion, the insertion of additional basic AAs in the polybasic CS compromised H4N2 replication and transmission in chickens, which were restored by reassortment with HPAIV H5N1. Therefore, due to the negative impact of the polybasic CS on virus fitness, the expansion of HPAIV H4N2 in nature is unlikely. Although it remains speculative, the evolution of natural HPAIV H4N2 will require prior reassortment with HPAIV H5N1-like gene segments to achieve a higher fitness followed by mutations in the HA to enable wide protease-activation. The fitness cost of the artificially induced polybasic CS as indicated by poor transmission and replication of H4N2 viruses carrying K^327^, R^327^ or H5N2-like HACS after ON inoculation may be a strong limiting factor for evolution of non-H5/H7 HPAIVs. Such viruses may occur as a result of error-prone RdRp activity but they are less fit than the wild-type H4N2 viruses and most likely will be eliminated from the quasispecies.

## 4. Materials and Methods

### 4.1. Viruses, Plasmids and Cells

A/quail/California/D113023808/2012 (H4N2) was kindly provided by Beate Crossley (The California Animal Health and Food Safety Laboratory System, Department of Medicine and Epidemiology, University of California, Davis, USA). Plasmids containing eight gene segments of HPAIV A/swan/Germany/R65/2006 (H5N1) were kindly provided by Jürgen Stech (Institute of Molecular Virology and Cell Biology, Friedrich-Loeffler-Institute (FLI), Greifswald-Insel Riems, Germany [11]). Plasmids containing eight gene segments of HPAIV A/chicken/Germany/AR1385/2015 (H7N7) were described [41]. pCAGGS plasmids encoding HAT and TMPRSS2 have been reported previously [50].

Primary chicken embryo kidney (CEK) cells used for the determination of replication kinetics were prepared according to standard procedures [51]. Madin–Darby canine kidney (MDCK), MDCK type II (MDCKII), and human embryonic kidney 293T (HEK293T) cell lines were obtained from the Cell Culture Collection in Veterinary Medicine of the FLI.

### 4.2. Generation of Plasmids and Recombinant Viruses

To generate the recombinant H4N2 virus (designated hereafter H4N2_wt) by reverse genetics, viral RNA was extracted using the QIAamp Viral RNA Mini Kit and transcribed into cDNA using the Omniscript RT Kit (Qiagen, Helden, Germany). All eight genomic segments of the H4N2 virus were amplified by specific primers and cloned into the pHW*SccdB* plasmid [52]. Using the HA encoding plasmid of H4N2_wt, three different CS motifs were generated by exchanging T^327^R or T^327^K, or by insertion of a polybasic CS resembling that of HPAIV A/chicken/Italy/8/1998 (H5N2) (designated hereafter H5N2-HACS) using the QuikChange II Site-Directed Mutagenesis Kit (Invitrogen, Carlsbad, CA, USA). Sequences of primers are available from the authors upon request.

Eight recombinant viruses (Table 1) were rescued in co-cultures of MDCKII and HEK293T cells as previously described [53]. In addition to the recombinant H4N2_wt, three recombinant H4N2 viruses carrying the HA4 with T^327^R (H4N2_T^327^R), T^327^K (H4N2_T^327^K) or H5N2-HACS (H4N2_H5N2-HACS) were constructed. Moreover, three recombinant H4N1 viruses carrying seven gene segments from H5N1 and HA from H4N2_wt (H5N1_HA4), HA4_T^327^R (H5N1_HA4_T^327^R) or HA4_T^327^K (H5N1_HA4_T^327^K) and one H4N7 virus carrying seven gene segments from H7N7 and the HA from H4N2_wt (H7N7_HA4) were successfully generated. Furthermore, HA of H4N2_wt, H4N2_T^327^R and H4N2_T^327^K H4N2 were cloned into the pCAGGS vector to increase protein expression.

### 4.3. Virus Propagation and Sequencing

Recombinant viruses were propagated in the allantoic sac of 10–11 day-old specific pathogen-free (SPF) embryonated chicken eggs (ECE) purchased from VALO BioMedia GmbH (Osterholz-Scharmbeck, Germany) according to the standard protocol of the World Organization for Animal Health (OIE) [54]. Inoculated eggs were examined daily and those with dead embryos were chilled at 4 °C and allantoic fluid (AF) was collected. AF was checked by a hemagglutination test using 1% chicken erythrocytes according to the OIE recommended protocol [54]. AF with a titer >16 (4log_2_) hemagglutination units was checked for bacterial contamination by streaking sheep blood agar at 37 °C for up to 72 h. Sterile AF was pooled and virus stocks were aliquoted and stored at −80 °C until use. All recombinant viruses with polybasic CS, except H4N2_wt, were handled in BSL3 facilities of the FLI. All gene segments of all viruses were sequenced to exclude unwanted mutations by Sanger sequencing using ABI BigDye Terminator v.1.1 Cycle Sequencing Kit (Applied Biosystems, Foster City, CA USA). The H4 AA numbering is based on the mature protein after removal of the signal peptide.

### 4.4. Replication Kinetics

CEK and MDCK cells were infected at a multiplicity of infection (MOI) of 0.001 in 12-well plates. After one hour at 37 °C and 5% CO_2_, the inoculum was removed, and the cells were incubated for two minutes with citric acid buffer (pH 3.0). The cells were washed twice with 1× phosphate buffered saline (PBS) and covered with minimum essential medium (MEM) containing 0.2% bovine serum albumin (BSA) (MP Biomedicals, Eschwege, Germany). Cells infected with recombinant H4N2_wt were grown in the presence or absence of 2 µg/µL of *N*-tosyl-l-phenyalanine chloromethyl ketone (TPCK)-treated trypsin (Sigma Aldrich, Steinheim, Germany). Plates were incubated at 37 °C and 5% CO_2_. Cells and supernatant were harvested at the indicated time points post-infection (hpi) and stored at −80 °C. Virus titers were determined by the plaque assay as described below. The replication kinetics were run in duplicates and repeated three times. Results are expressed as average values and the standard deviation was indicated for all replicates.

### 4.5. Plaque Assay

Confluent MDCKII or MDCK cells in 6-well plates were washed once with PBS and incubated with 10-fold serial dilutions of propagated viruses or samples for 1 hour at 37 °C in a 5% CO_2_ atmosphere. Thereafter, cells were washed twice with 1× PBS and covered by semi-solid Bacto^TM^ Agar (BD, Pont-de-Claix, France) with 50% MEM containing 4% BSA (MP Biomedicals, Eschwege, Germany). All plates were incubated for 3 days at 37 °C in 5% CO_2_. In MDCKII cells, TPCK-treated trypsin (2 µg/mL) was added to cells infected with H4N2_wt and H4N2_H5N2-HACS. Moreover, cell-to-cell spread of all indicated viruses in MDCK cells was studied with or without exogenous TPCK-treated trypsin. Cells were fixed using 10% formaldehyde containing 0.1% crystal violet for at least 48 h. Plaques were counted and viral titers were expressed as plaque-forming units per ml (PFU/mL). Moreover, to determine cell-to-cell spread of different viruses, the size of at least 50 plaques obtained for each virus was measured by microscopy (Eclipse Ti-S with software NIS-Elements, version 4.0; Nikon, Amsterdam, Netherlands). The diameter of plaques of the H4N2_wt in the absence of trypsin was adjusted to 100%. The plaque size obtained by different recombinant viruses relative to the H4N2_wt was calculated.

### 4.6. Western Blot

HA cleavability was assessed in HEK293T cells in the presence or absence of exogenous proteases (i.e., trypsin, TMPRSS2 and HAT) using standard Western Blot procedures with few modifications [55]. The cleavage of HA of H4N2_wt, H4N2_T^327^R and H4N2_T^327^K in the presence or absence of 2 µg/mL TPCK-treated trypsin was studied by transfecting cells with 5 µg pCAGGS plasmid coding for HA of the different viruses using Lipofectamine 2000 transfection reagent (ThermoFischer Scientific, Karlsruhe, Germany). The transfected cells were incubated with MEM containing 0.2% BSA at 37 °C in 5% CO_2_ for 24 h. For TMPRSS2 and HAT, HEK293T cells that do not express an endogenous HAT or TMPRSS2 [56], were co-transfected with 1 µg pCAGGS plasmids containing H4N2_T^327^K, as well as 10 ng plasmid coding for each protease in the presence or absence of 50 µM furin inhibitior MI-1148 (kindly provided by Torsten Steinmetzer, Institute of Pharmaceutical Chemistry, Philipps-University Marburg, Marburg, Germany) [50]. After 48 h, transfected cells were harvested, washed with PBS and sedimented at 14,000× *g* for 15 min. Proteins were denatured in Laemmli buffer for 5 min at 99 °C. Proteins, as well as a stained protein marker, were separated by discontinuous sodium dodecyl sulfate-10% polyacrylamide gel electrophoresis (SDS-PAGE). Proteins were transferred to nitrocellulose membranes using a blotting device at 25 V for 2 h and blots were blocked for 1 h in 5% skimmed milk. For the detection of the HA protein, polyclonal specific anti-H4N2-HA2 antibodies were generated in rabbits. The β-Actin as internal control was detected using monoclonal antibodies. All blots were incubated with the primary antibodies overnight at 4 °C. Bound primary antibodies were detected by the incubation of blots with peroxidase-conjugated anti-rabbit IgG for HA or anti-mouse IgG antibodies (Jackson Immuno Research, Cambridgeshire, UK) for β-Actin. The immunodetection was done by chemiluminescence using Clarity^TM^ Western ECL Substrate (BioRad, Feldkirchen , Germany). Images were captured by a Bio-Rad Versadoc 4000 Molecular Imager (BioRad, Munich, Germany) and Quantity One software (BioRad, Munich, Germany).

### 4.7. Animal Experiments

All animal experiments were carried out according to the German Regulations for Animal Welfare in the biosafety level-3 (BSL3) animal facilities of the FLI after approval by the authorized ethics committee of the State Office of Agriculture, Food Safety and Fishery in Mecklenburg–Western Pomerania (LALLF M-V, permission number 7221.3-1.1-051-12). The commissioner for animal welfare at the FLI representing the Institutional Animal Care and Use Committee (IACUC) approved all experiments (TV04/17; December 2016).

SPF eggs from white leghorn chickens were purchased from VALO BioMedia GmbH (Osterholz-Scharmbeck, Germany) and incubated at the animal quarantine facilities of the FLI until hatch. Male and female chickens, at 6- to 8-weeks-old, were allocated into different groups and infected via the oculo-nasal (ON) or intravenous (IV) routes. To determine the virulence of recombinant viruses via the ON route, chickens were inoculated with 0.2 mL containing 10^5^ PFU per bird (~0.1 mL in each side). One day post-inoculation (dpi), sentinel chickens were added to assess bird-to-bird transmission. To determine the IVPI of indicated viruses, 10 birds were injected via the cutaneous ulnar vein with 0.1 mL 1:10 diluted AF according to the OIE standard protocol [54]. All birds were observed daily for clinical signs and mortality for 10 (IV) or 14 (ON) dpi. The severity of clinical signs was assessed using a standard pathogenicity index (PI) as recommended [54]. Briefly, healthy birds were scored with 0. Birds showing one clinical sign (e.g., ruffled feather, depression, nervous signs, diarrhea, edema, hemorrhages or cyanosis in the unfeathered parts like shanks, comb or wattle) were given score 1, and birds exhibiting at least two clinical signs were scored with 2. Dead birds were given score 3 until the termination of the experiment. Severely diseased birds were euthanized and scored as dead on the next observation day. The PI was calculated using the sum of daily arithmetic means of all birds divided by ten or 14 (number of observation days) in each group. The PI value ranged from 0 (avirulent) to 3 (highly virulent).

Oropharyngeal and cloacal swabs were collected at 4 dpi using MEM containing antibiotics. Virus excretion in swab samples was determined using NucleoSpin 8/96 PCR Clean-up Core Kit (Macherey & Nagel, Düren, Germany) according to the manufacturer instructions using the TECAN Freedom EVO System (TECAN, Männedorf, Switzerland). After RNA extraction the viral load in the swab samples was assessed by generic real-time-reverse-transcription polymerase chain reaction (RT-qPCR) targeting the AIV Matrix gene [57]. Each RT-qPCR run included standard curves generated by serial dilutions of H4N2 or H5N1 virus. The amount of RNA was determined by plotting the CT-value of a given sample against the dilution in standard curves and expressed as viral RNA copies/mL. Results of each group are expressed as arithmetic mean and standard deviation of virus titers in oropharyngeal and cloacal swabs.

At the end of the observation period, all surviving birds were euthanized by Isoflurane^®^ (CP-Pharma, Burgdorf, Germany) inhalation and blood was collected. Sera were tested for anti-AIV NP antibodies using ID screen Influenza Antibody Competition Multispecies kit (IDvet, Montpellier, France) according to the manufacturer recommendations.

### 4.8. Histopathology and Immunohistochemistry

The severity of pathohistological lesions and distribution of recombinant viruses in the trachea, lungs, heart, liver, pancreas, kidneys, thymus, spleen, proventriculus, gizzard, duodenum, jejunum, caecum, bursa of Fabricius and brain from at least two inoculated birds per group was analyzed at 4 dpi except for chickens inoculated with H5N1_HA4_T^327^K, which died at 2 dpi and were kept in the refrigerator. Organ samples were fixed immediately in 10% neutral buffered formalin. After processing, the samples were embedded in paraffin wax, sectioned at 2–4 µm, stained with hematoxylin and eosin, and screened for histopathological changes. The severity of necrotizing inflammation and lymphatic depletion was scored blind on an ordinal 0 to 3 scale: 0 = no change; 1 = mild; 2 = moderate, and 3 = severe necrosis or lymphatic depletion. Following sections were used for immunohistochemistry using the avidin–biotin–peroxidase complex method (Vector Laboratories Burlingame, CA, USA) with a primary polyclonal rabbit anti-NP antibody (1:750), and a secondary biotinylated goat anti-rabbit IgG (Vector Laboratories, Burlingame, CA, USA) antibody (1:200) as described [58,59]. The distribution of NP antigen in the endothelium and parenchyma was blind semiquantitatively scored on an ordinal 0 to 3 scale: 0 = negative; 1 = focal or oligofocal, 2 = multifocal, and 3 = coalescing to diffuse immunoreactive cells.

### 4.9. Statistics

Statistical differences for replication kinetics in CEK and MDCK cells were analyzed using ordinary one-way ANOVA with post hoc Tukey tests. Plaque size in MDCKII and MDCK cells and RT-qPCR results of oropharyngeal shedding 4 dpi were evaluated using ordinary one-way ANOVA with Bonferroni correction to H4N2_wt. A *p*-value of <0.05 was considered significant. All analyses were done using GraphPad Prism 8 software (CA, USA).

### 4.10. Biosafety

All recombinant DNA protocols were approved by the State Office for Health and Social Affairs of Western Pomerania (LAGuS MV-AZ: 6/08-2/96). Gain-of-function experiments were approved by the German Research Foundation (DFG: VE 780/1) and by the biorisk committee of the Friedrich-Loeffler-Institut (FLI) before starting the project. Experiments with HA specifying polybasic cleavage sites were done in the biosafety level (BSL) 3 laboratory and animal facilities at the FLI. All work was done by experienced researchers who participated in the Project Manager Course for Genetic Engineering and followed the regulations for handling genetically modified organisms.

## Figures and Tables

**Table 1:** Recombinant viruses generated in this study.

Virus	Cleavage Site	Source of	
		HA	Other Gene Segments
H4N2_wt	^322^PEKRRTR/G^329^	H4N2	H4N2
H4N2_HA_T^327^R	^322^PEKRR**R**R/G^329^	H4N2	H4N2
H4N2_HA_T^327^K	^322^PEKRR**K**R/G^329^	H4N2	H4N2
H4N2_H5N2-HACS *	^322^P**QRRREKK**R/G^331^	H4N2	H4N2
H7N7_HA4	^322^PEKRRTR/G^329^	H4N2	H7N7
H5N1_HA4	^322^PEKRRTR/G^329^	H4N2	H5N1 (Gs/Gd)
H5N1_HA4_T^327^R	^322^PEKRR**R**R/G^329^	H4N2	H5N1 (Gs/Gd)
H5N1_HA4_T^327^K	^322^PEKRR**K**R/G^329^	H4N2	H5N1 (Gs/Gd)

Residues in bold indicate the point mutations or insertion compared to the wild type H4N2 virus cleavage site ^322^PEKRRTR/G^329^; HACS = hemagglutinin cleavage site. * Trials to generate an H4N2 carrying a HPAIV H5N2 HACS (^322^PQ**RRRKKR**/G^330^) were not successful. After several rescue attempts, a virus was obtained with a spontaneous insertion of glutamic acid (^322^PQ**RRR**E**KKR**/G^331^) in the HACS. Gs/Gd = Goose Gunagdong H5N1 lineage.

**Table 2:** Results of the clinical examination after the challenge of chickens with different recombinant viruses in this study.

Virus	Oculonasal					IVPI
	Inoculated Chickens			Contact Chickens		
	PI ^1^	Mortality (MDT; Range)	SC	Mortality (MDT; Range)	SC	
H4N2_wt	0.0	0/6 (n.a.; n.a.)	4/4	0/3 (n.a.; n.a.)	3/3	n.d.
H4N2_HA_T^327^R	0.0	0/6 (n.a.; n.a.)	4/4	0/4 (n.a.; n.a.)	4/4	0.0
H4N2_HA_T^327^K	0.0	0/6 (n.a.; n.a.)	4/4	0/4 (n.a.; n.a.)	1/4	0.6
H4N2_H5N2-HACS ^2^	0.0	0/6 (n.a.; n.a.)	3/4	0/4 (n.a.; n.a.)	0/4	0.1
H7N7_HA4_wt	0.0	0/6 (n.a.; n.a.)	4/4	0/4 (n.a.; n.a.)	4/4	0.3
H5N1_HA4_wt	0.5	0/6 (n.a.; n.a.)	4/4	0/4 (n.a.; n.a.)	4/4	2.1
H5N1_HA4_T^327^R	2.4	6/6 (3.8; 3–4)	n.a.	3/4 (6.0; 4–8)	1/1	n.d.
H5N1_HA4_T^327^K	2.7	6/6 (2.0; 2)	n.a.	4/4 (3.5; 3–4)	n.a.	2.8

^1^ PI = pathogenicity index, mortality rate = number of dead birds/total number of birds per group, MDT = mean death time and range of days with mortality after inoculation or adding the sentinel birds, SC = seroconversion using an NP-specific ELISA showing number of positive birds/total examined, IVPI = intravenous pathogenicity index, n.a. = not applicable, n.d. = not done. ^2^ HACS = hemagglutinin cleavage site.
